# The Value of Carotid Artery Plaque and Intima‐Media Thickness for Incident Cardiovascular Disease: The Multi‐Ethnic Study of Atherosclerosis

**DOI:** 10.1161/JAHA.113.000087

**Published:** 2013-04-24

**Authors:** Joseph F. Polak, Moyses Szklo, Richard A. Kronmal, Gregory L. Burke, Steven Shea, Anna E. H. Zavodni, Daniel H. O'Leary

**Affiliations:** 1 Ultrasound Reading Center, Department of Radiology, Tufts Medical Center, Boston, MA (J.F.P.); 2 Department of Epidemiology, Johns Hopkins University, Baltimore, MD (M.S.); 3 Collaborative Health Studies Coordinating Center, University of Washington, Seattle, WA (R.A.K.); 4 Division of Public Health Sciences, Wake Forest School of Medicine, Winston‐Salem, NC (G.L.B.); 5 Departments of Medicine and Epidemiology, Columbia University, New York, NY (S.S.); 6 Department of Medical Imaging, University of Toronto, Ontario, Canada (A.H.Z.); 7 Saint Elizabeth's Medical Center, Boston, MA (D.H.L.)

**Keywords:** cardiovascular diseases, carotid arteries, coronary disease, stroke

## Abstract

**Background:**

Carotid artery plaques are associated with coronary artery atherosclerotic lesions. We evaluated various ultrasound definitions of carotid artery plaque as predictors of future cardiovascular disease (CVD) and coronary heart disease (CHD) events.

**Methods and Results:**

We studied the risk factors and ultrasound measurements of the carotid arteries at baseline of 6562 members (mean age 61.1 years; 52.6% women) of the Multi‐Ethnic Study of Atherosclerosis (MESA). ICA lesions were defined subjectively as >0% or ≥25% diameter narrowing, as continuous intima‐media thickness (IMT) measurements (maximum IMT or the mean of the maximum IMT of 6 images) and using a 1.5‐mm IMT cut point. Multivariable Cox proportional hazards models were used to estimate hazard ratios for incident CVD, CHD, and stroke. Harrell's C‐statistics, Net Reclassification Improvement, and Integrated Discrimination Improvement were used to evaluate the incremental predictive value of plaque metrics. At 7.8‐year mean follow‐up, all plaque metrics significantly predicted CVD events (n=515) when added to Framingham risk factors. All except 1 metric improved the prediction of CHD (by C‐statistic, Net Reclassification Improvement, and Integrated Discrimination Improvement. Mean of the maximum IMT had the highest NRI (7.0%; *P*=0.0003) with risk ratio of 1.43/mm; 95% CI 1.26–1.63) followed by maximum IMT with an NRI of 6.8% and risk ratio of 1.27 (95% CI 1.18–1.38).

**Conclusion:**

Ultrasound‐derived plaque metrics independently predict cardiovascular events in our cohort and improve risk prediction for CHD events when added to Framingham risk factors.

## Introduction

Atherosclerotic plaques that develop in the different arterial beds of the human body are the underlying pathologic process resulting in incident cardiovascular events.^1–6^

By ultrasound, atherosclerotic plaque can be defined as a measured protrusion of the intima‐media thickness (IMT) of >1.5 mm into the lumen^7–8^ or by a subjective estimate.^7,9–10^

Recent studies have suggested that the presence of plaque, defined subjectively or as a local thickening of >1.5 mm, might be predictive of cardiovascular outcomes.^8,11^ We propose to compare different ultrasound measures of carotid artery plaque and study their associations with incident cardiovascular disease (CVD), coronary heart disease (CHD), and stroke.

We study 6 different published definitions of plaque: subjectively defined as present,^10^ subjectively causing a relative narrowing >25%,^12^ measured as a continuous IMT value in the internal carotid artery (ICA),^13^ combining a subjective judgment of being present or far wall IMT >1.5 mm in the common carotid artery and ICA,^7,11^ measured as a maximum ICA IMT >1.5 mm,^8^ and calculated as the mean of the maximum ICA IMT.^14–15^

We hypothesize that IMT and subjective‐based definitions of plaque are predictive of cardiovascular outcomes. We study this hypothesis and compare the predictive value of these different plaque measurements in the Multi‐Ethnic Study of Atherosclerosis (MESA).

## Materials and Methods

### Population

The MESA is a multiethnic population of 6814 men and women aged 45 to 84 years without evidence of clinical CVD at baseline enrolled between July 2000 and August 2002 at 6 sites in the United States. The MESA cohort includes white, African American, Hispanic, and Chinese participants. Participants were excluded if they had physician diagnosis of heart attack, stroke, transient ischemic attack, heart failure, angina, atrial fibrillation, a history of any cardiovascular procedure, weight >300 lb, pregnancy, or any medical condition that would prevent long‐term participation.^16^ MESA protocols and all studies described herein have been approved by the institutional review boards of all collaborating institutions.

### Risk Factors and Anthropomorphic Variables

Age, gender, race/ethnicity, and medical history were self‐reported. Use of antihypertensive and lipid‐lowering medications was also recorded. Current smoking was defined as self‐report of ≥1 cigarettes in the past 30 days. Resting systolic and diastolic blood pressures (BPs) were measured in the seated position using a Dinamap model Pro 100 automated oscillometric sphygmomanometer (Critikon).

Glucose and lipids were measured after a 12‐hour fast. Serum glucose was measured by rate reflectance spectrophotometry on the Vitros analyzer (Johnson & Johnson Clinical Diagnostics, Inc). Diabetes mellitus was determined according to the 2003 American Diabetes Association fasting criteria.^17^ Total cholesterol was measured using a cholesterol oxidase method (Roche Diagnostics), as was high‐density lipoprotein (HDL) after precipitation of non‐HDL cholesterol with magnesium/dextran.

### Carotid Artery Measures

The patients were supine with their head rotated 45° toward the side opposite to the side being imaged. A transverse sweep was recorded from the low neck through the carotid artery bifurcation into the ICA. Doppler velocity measurements were made at the site of any bulb or proximal ICA lesion or in the proximal ICA if no lesions were seen. The common carotid artery (CCA) was then imaged at 45° from the vertical with the beginning of the bulb shown to the left of the image. Three views centered on the ICA bulb were taken: 1 anterior, 1 lateral (at 45°), and 1 posterior. A matrix array probe (M12L, General Electric) was used with the frequency set at 13 MHz for the CCA and 9 MHz for the ICA and with 2 focal zones at a frame rate of 32 frames/s.

All carotid artery measurements were blinded and made at the ultrasound reading center in Boston, Massachusetts. IMT measurements were made on near and far walls of the CCA (1 projection) and the ICA IMT measurements were centered on the bulb (3 projections) using hand‐drawn continuous tracings of the intima–lumen and media–adventitia interfaces, which were then processed by use of a previously described algorithm.^18^ All videotaped images were reviewed for the presence and severity of any lesion, in either the near or far walls. Mean of the maximum CCA IMT was calculated as the mean of the maximum IMT on the near and far walls of the right and left CCAs. The presence of lesions was graded with a semiquantitative scale of 0% (absent lesion) and lesions causing relative narrowing of 1% to 24% and 25% to 49%.^10^ Doppler velocity measurements at the site of the lesions with peak‐systolic velocities ≥125 cm/s were considered to be >50% diameter narrowing.^19^ The greater values of the right and left sides were used for final grading of lesion severity. Readers were reviewed every 2 to 3 weeks during sessions to establish uniformity of interpretations by having the principal investigator (J.F.P.) review selected studies.

Blinded replicate scans were performed on 144 participants with the same readers performing the blinded reread.

Variables of interest were:

1. Presence of plaque (>0% as graded)^10^
2. Presence of plaque (Framingham Heart Study: ≥25% diameter narrowing)^12^
3. Maximum of the ICA IMT on either the right or left side^13^
4. Presence of plaque or maximum of the far wall IMT >1.5 mm in either the ICA or CCA^7,11^
5. Maximum of >1.5 mm in the ICA^8^
6. Mean of the maximum IMT of the ICA^14–15^

### Outcomes

Events were identified during follow‐up examinations and by telephone interview conducted every 9 to 12 months to inquire about all interim hospital admissions, cardiovascular outpatient diagnoses, and deaths. Copies were obtained of all death certificates and of all medical records for hospitalizations and outpatient cardiovascular diagnoses. Two physicians from the MESA study events committee independently reviewed all medical records for end point classification and assignment of incidence dates. The review process included all generated *International Classification of Disease* definitions but the final adjudication of MESA end points was based on specific criteria applied to data obtained from medical records by 2 committee members or by the whole study events committee in case of disagreement.

Three end points were used in these analyses. CHD events included myocardial infarction (MI), death due to CHD, resuscitated cardiac arrest, definite or probable angina followed by coronary revascularization, and definite angina not followed by coronary revascularization. Cases of coronary artery revascularization that did not have a concurrent diagnosis of angina were not included.

The diagnosis of MI was based on a combination of symptoms, electrocardiographic findings, and circulating cardiac biomarkers. Death was considered related to CHD if it occurred within 28 days after an MI, if the participant had experienced chest pain within the 72 hours preceding death, or if the participant had a history of CHD and died without documentation of any other cause of death. Resuscitated cardiac arrest included participants who successfully recovered from full cardiac arrest through cardiopulmonary resuscitation.

Adjudicators graded the presence of angina based on the following criteria. A classification of definite or probable angina required clear and definite documentation of symptoms without the development of MI. Definite angina also required objective evidence of reversible myocardial ischemia or obstructive coronary artery disease.

Stroke was based on the rapid onset of a documented focal neurologic deficit lasting 24 hours or until death or, if <24 hours, with accompanying evidence of a clinically relevant lesion on brain imaging. Patients with focal neurologic deficits secondary to brain trauma, tumor, infection, or other nonvascular cause were excluded.

Cardiovascular events were defined as CHD, stroke (not transient ischemic event), or death following a diagnosed stroke. A more detailed description of the MESA follow‐up methods is available at http://www.mesa-nhlbi.org.

### Statistical Analyses

The mean (and SD) values of continuous variables and the distribution of dichotomous variables as a percentage in each group are shown for the participants.

Reproducibility for dichotomous variables was reported as κ values and for continuous measurements as correlation coefficients with estimated 95% CIs.

A baseline multivariable Cox proportional hazards model regression model was created with the components of the Framingham risk score: age, gender, systolic BP, diabetes, HDL‐cholesterol, total cholesterol, smoking, and BP‐lowering therapy.^20^ Calculated Framingham risk scores were not directly used due to possible issues of the applicability to different ethnic groups.^21^ The components of the respective Framingham risk factors were considered as separate independent predictors. All analyses were adjusted for race/ethnicity.

Analyses were repeated for the 2 components of CVD: CHD and stroke events. The model for CHD events included the original risk factors described by Wilson et al.^22^ The risk factors used in the general cardiovascular risk model as reported by D'Agostino et al^20^ were used for CVD and stroke events.

Models with each of the a priori defined plaque metrics were added to the baseline model and the hazards ratio for the plaque metric obtained. Harrell's C‐statistics were obtained, and the predictive value of each plaque metric was evaluated by comparing the model with the plaque metric to the baseline model using the differences in Harrell's C‐statistic. The proportional hazards assumption was verified using Schoenfeld's residuals. Net Reclassification Improvement (NRI) and Integrated Discrimination Improvement (IDI) were calculated as described by Pencina et al.^23–24^ NRI was calculated from the Framingham predicted risk cut points of 6% and 20% at 10 years,^8,23^ translating into 4.7% and 15.6% at a mean follow‐up of 7.8 years.

Sensitivity analyses were performed by adding common carotid IMT to the respective models.

Statistical analyses were performed using JMP 9.0 (SAS institute Inc) and STATA 11.2 (StataCorp). Level of statistical significance was set at *P*≤0.05. NRI and IDI were calculated with the help of a STATA add‐on from the Uppsala Clinical Research Center (http://www.ucr.uu.se/en/index.php/epistat/program-code/306-nri-and-idi).

Dr. Polak had full access to all the data in the study and takes responsibility for the integrity of the data and the accuracy of the data analysis.

## Results

Of the 6814 MESA participants, 6562 had complete data for risk factors and carotid artery measurements at the baseline examination. Data were missing on smoking status (n=22), diabetes status (n=24), systolic BP (n=3), HDL‐cholesterol levels (n=26), subjective grading of carotid plaque (n=98), and ICA IMT measurements (n=185). Seventy‐five individuals did not have a carotid examination. Individuals excluded from the analysis because of missing data were more likely to be men, have a higher prevalence of diabetes, and more likely to be on antihypertensive therapy. Event rates for CVD, CHD, and stroke were not significantly different between excluded and nonexcluded individuals.

Table 1 summarizes the characteristics of the population studied with a mean follow‐up of 7.8 years. Average age was 61.1 years and 47.4% of the population was male. There were 38.7% whites, 12.1% Chinese, 27.3% black, and 21.9% Hispanic participants. Mean±SD systolic BP was 126.5±21.5 mm Hg, HDL‐cholesterol was 51.0±14.8 mg/dL, and total cholesterol was 194.2±35.7 mg/dL. The prevalence of diabetes was 13.9%, and 13.1% of participants were current smokers. Prevalent plaque ranged from 13.2% to 43.5% depending on the definition used. During the follow‐up, 5.7% developed incident CHD, and 2.1%, incident stroke.

**Table 1.** Demographics of the Cohort Participants With Risk Factor Values and Plaque Measurements, Multi‐Ethnic Study of Atherosclerosis (2000–2002)

Variable	Value*
Age, y	61.1±10.2
Sex (men/women)	47.4% (3110/3452)
Ethnicity	
White	38.7% (2541)
Chinese	12.1% (792)
Black	27.3% (1790)
Hispanic	21.9% (1439)
Systolic blood pressure, mm Hg	126.5±21.5
HDL‐cholesterol, mg/dL	51.0±14.8
Total cholesterol, mg/dL	194.2±35.7
Diabetes (yes/no)	13.9% (911/5651)
Current smoker (yes/no)	13.1% (860/5702)
Any plaque above 0% (yes/no)	41.9% (2748/3814)
Plaque above 25% (yes/no)	13.2% (863/5699)
Maximum internal carotid artery IMT, mm	1.595±1.038
Combined subjective and far wall IMT >1.5 mm (yes/no)	43.5% (2855/3706)
Maximum ICA IMT >1.5 mm (yes/no)	38.5% (2526/4036)
Mean of the maximum ICA IMT, mm	1.074±0.605
Incident cardiovascular disease (yes/no)	7.9% (515/6047)
Incident coronary heart disease (yes/no)	5.7% (372/6190)
Incident stroke (yes/no)	2.1% (139/6423)

HDL indicates high‐density lipoprotein; IMT, intima‐media thickness; ICA, internal carotid artery.

* Mean±SD for continuous variables and percent values (numerical values) for categorical variables.

Reproducibility estimates are shown in Table 2. The dichotomous variables showed interscan κ values ranging from 0.833 to 0.944. Paired correlation coefficients ranged from 0.90 to 0.91.

**Table 2.** Paired Replicate Rescan Estimates of Variability of the Different Plaque Metrics in the Multi‐Ethnic Study of Atherosclerosis

Methods of Estimating Plaque	Value	Lower 95% CI	Upper 95% CI	No. of Replicate Values	Measure of Agreement/Association
Any plaque (>0%)	0.886	0.854	0.919	144	κ
Plaque (≥25%)	0.833	0.793	0.874	144	κ
Maximum internal carotid artery IMT*, mm	0.908	0.874	0.933	141	Pearson
Combined subjective and far wall IMT >1.5 mm	0.944	0.917	0.971	144	κ
Maximum ICA IMT (>1.5 mm)*	0.870	0.836	0.904	141	κ
Mean of the maximum ICA IMT, mm	0.897	0.859	0.925	141	Pearson

Examinations were blindly reread after blinded replicate scans. Because of the blinded nature of the replicate scanning protocol, the same reader was likely to reread the replicate examination. From a total of 151 blinded rescans and then blinded interpretations, 144 were from the same readers. ICA indicates internal carotid artery; IMT, intima‐media thickness.

* Measurements were not obtained for the ICA IMT in 3 of the 144 replicate studies.

Unadjusted and adjusted Kaplan–Meier event rates are shown in the Figure.

**Figure 1.**
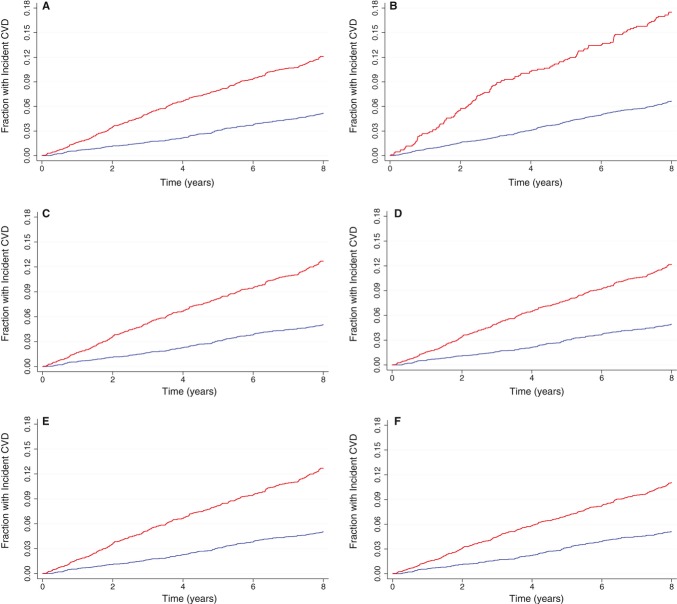
Kaplan‐Meier curves depicting the probability of developing a cardiovascular event as a function of time for the different plaque metrics (absence of plaque is the blue line; presence of plaque is the red line): (A) any plaque,^10^ (B) stenosis ≥25%,^12^ (C) median of maximum ICA IMT,^13^ (D) any plaque or maximum IMT >1.5 mm,^7,11^ (E) maximum ICA IMT >1.5 mm,^8^ and (F) median of mean of maximum IMT.^15^ ICA indicates internal carotid artery; IMT, intima‐media thickness; CVD, cardiovascular disease.

Results for multivariable Cox proportional hazards models for CVD, CHD, and stroke as outcomes are shown in Table 3. All Framingham risk factors predicted CVD and CHD events, while gender and use of BP‐lowering medications were not independent predictors of stroke events. In the base model, C‐statistics were 0.743 (95% CI 0.724–0.762) for CVD, 0.729 (95% CI 0.705– 0.752) for CHD, and 0.774 (95% CI 0.739– 0.809) for stroke.

**Table 3.** Results of Multivariable Cox Proportional Hazards Model With Separate Outcomes: CVD, CHD, and Stroke

Variable	CVD*				CHD*				Stroke*			
	HR	HR Lower 95% CI	HR Upper 95% CI	*P*‐Value	HR	HR Lower 95% CI	HR Upper 95% CI	*P*‐Value	HR	HR Lower 95% CI	HR Upper 95% CI	*P*‐Value
Age, y	1.06	1.04	1.07	<0.001	1.05	1.04	1.06	<0.001	1.06	1.03	1.08	<0.001
Sex (men)	1.76	1.45	2.15	<0.001	2.06	1.63	2.61	<0.001	1.09	0.75	1.58	0.66
Current smoker (yes)	1.97	1.55	2.50	<0.001	1.72	1.29	2.28	<0.001	2.08	1.32	3.29	0.002
Diabetes (yes)	1.59	1.36	2.09	<0.001	1.75	1.37	2.23	<0.001	1.85	1.23	2.79	0.003
Systolic blood pressure, mm Hg	1.013	1.009	1.018	<0.001	1.012	1.007	1.017	<0.001	1.020	1.013	1.028	<0.001
Cholesterol, mg/dL	1.005	1.003	1.007	<0.001	1.005	1.002	1.008	<0.001	1.005	1.001	1.009	0.02
HDL cholesterol, mg/dL	0.986	0.979	0.994	<0.001	0.987	0.978	0.996	0.004	0.982	0.969	0.994	0.005
Blood pressure–lowering medications (yes)	1.39	1.14	1.68	0.001					1.42	0.96	2.09	0.08

Plaque metrics are not included. Race/ethnicity was significant for effect at *P*=0.04 for CVD, at *P*=0.02 for CHD, and nonsignificant for stroke. CVD indicates cardiovascular disease; CHD, coronary heart disease; HR, hazard ratio; HDL, high‐density lipoprotein.

* Model for CVD events has a C‐statistic of 0.743 (95% CI 0.724–0.762).

* Model for CHD events has a C‐statistic of 0.729 (95% CI 0.705–0.752).

* Model for Stroke a C‐statistic of 0.774 (95% CI 0.739–0.809).

The results of adding each of the selected plaque metrics to the Cox proportional hazards model for CVD are presented in Table 4. All plaque parameters were significantly associated with incidence of CVD in multivariable adjusted analyses. Hazard ratios varied from 1.21 to 1.65, with the strongest association seen for plaque ≥25% and the weakest for mean of the maximum ICA IMT (hazard ratio=1.21). All of the plaque metrics were associated with a significant increase in C‐statistic when added to the baseline Cox proportional hazards model. The NRI was significant only for the mean of the maximum internal carotid artery IMT at 5.2%, whereas the IDI was largest for maximum ICA IMT.

**Table 4.** Respective HRs, Change in C‐Statistic, NRI and IDI for Cardiovascular Disease Events When Each Plaque Metric is Added to a Baseline Model With Risk Factors Shown in Table 3

Variable	HR	HR Lower 95% CI	HR Upper 95% CI	*P*‐Value	Change in C‐Statistic	Lower 95% CI	Upper 95% CI	*P*‐Value	NRI (%)	*P*‐Value	IDI	*P*‐Value
Plaque >0%	1.45	1.20	1.76	<0.001	0.0046	−0.0003	0.0094	0.065	2.0	0.201	0.0022	0.01
Plaque ≥25%	1.65	1.34	2.03	<0.001	0.0059	0.0009	0.0109	0.02	2.1	0.201	0.0056	<0.001
Maximum of the ICA IMT, mm	1.21	1.13	1.30	<0.001	0.0068	0.0016	0.0120	0.01	2.9	0.079	0.0062	<0.001
Plaque or maximum of the far wall IMT >1.5 mm	1.49	1.22	1.82	<0.001	0.0057	0.0005	0.0108	0.033	2.7	0.107	0.0023	0.01
Maximum of the ICA IMT >1.5 mm	1.48	1.21	1.80	<0.001	0.0053	0.0002	0.0104	0.042	3.2	0.060	0.0022	0.02
Mean of the maximum ICA IMT, mm	1.33	1.18	1.49	<0.001	0.0065	0.0018	0.0112	0.007	5.2	0.002	0.0049	<0.001

HR indicates hazard ratio; NRI, net reclassification improvement; IDI, integrated discrimination improvement; ICA, internal carotid artery; IMT, intima‐media thickness.

The results of adding each of the selected plaque metrics to the Cox proportional hazards model for CHD are presented in Table 5. All plaque parameters were significantly associated with incidence of CHD in multivariable adjusted analyses. Hazard ratios varied from 1.27 to 1.80, with the strongest association seen for presence of an IMT >1.5 mm. All of the plaque metrics were associated with a significant increase in C‐statistic when added to the baseline Cox proportional hazards model. The NRI was significant for all metrics except for the maximum ICA IMT >1.5 mm. The largest NRI was for mean of the maximum IMT at 7.0%, followed by the maximum ICA IMT at 6.8%, whereas the IDI was largest for maximum ICA IMT, followed by maximum ICA IMT >1.5 mm.

**Table 5.** Respective HRs, Change in C‐Statistic, NRI and IDI for Coronary Heart Disease Events When Each Plaque Metric Is Added to a Baseline Model With Risk Factors Shown in Table 3

Variable	HR	HR Lower 95% CI	HR Upper 95% CI	*P*‐Value	Change in C‐Statistic	Lower 95% CI	Upper 95% CI	*P*‐Value	NRI (%)	*P*‐Value	IDI	*P*‐Value
Plaque >0%	1.67	1.33	2.10	<0.001	0.0089	0.0005	0.0173	0.037	4.2	0.035	0.0035	<0.001
Plaque ≥25%	1.67	1.30	2.13	<0.001	0.0078	0.0012	0.0145	0.021	5.0	0.0053	0.0037	0.003
Maximum of the ICA IMT, mm	1.27	1.18	1.38	<0.001	0.0123	0.0042	0.0203	0.003	6.8	0.0005	0.0074	<0.001
Plaque or maximum of the far wall IMT >1.5 mm	1.77	1.40	2.25	<0.001	0.0111	0.0018	0.0204	0.019	4.7	0.032	0.0045	<0.001
Maximum of the ICA IMT >1.5 mm	1.80	1.42	2.27	<0.001	0.0109	0.0014	0.0205	0.025	3.9	0.062	0.0053	<0.001
Mean of the maximum ICA IMT, mm	1.43	1.26	1.63	<0.001	0.0131	0.0056	0.0207	0.001	7.0	0.0003	0.0035	<0.001

HR indicates hazard ratio; NRI, net reclassification improvement; IDI, integrated discrimination improvement; ICA, internal carotid artery; IMT, intima‐media thickness.

The results of adding each of the selected plaque metrics to the Cox proportional hazards model for stroke are presented in Table 6. Only carotid plaque causing >25% diameter narrowing at the bulb was significantly associated with incident stroke in multivariable adjusted analyses with a hazard ratio of 1.60. None of the plaque metrics were associated with a significant increase in C‐statistic when added to the baseline Cox proportional hazards model. The NRI values were not significant for all metrics. The IDI was significant for presence of plaque causing >25% diameter narrowing at the carotid artery bulb.

**Table 6.** Respective HRs, Change in C‐Statistic, NRI and IDI for Stroke Events When Each Plaque Metric Is Added to a Baseline Model With Risk Factors Shown in Table 3

Variable	HR	HR Lower 95% CI	HR Upper 95% CI	*P*‐Value	Change in C‐Statistic	Lower 95% CI	Upper 95% CI	*P*‐Value	NRI (%)	*P*‐Value	IDI	*P*‐Value
Plaque >0%	1.11	0.76	1.62	0.59	0.0010	−0.0013	0.0033	0.85	1.4	0.32	−0.00002	0.90
Plaque ≥25%	1.60	1.08	2.35	0.02	0.0039	−0.0044	0.0122	0.93	1.4	0.63	0.00253	0.017
Maximum of the ICA IMT, mm	1.11	0.95	1.29	0.18	0.0014	−0.0035	0.0064	0.85	2.4	0.21	0.00066	0.20
Plaque or maximum of the far wall IMT >1.5 mm	1.15	0.78	1.70	0.49	0.0013	−0.0017	0.0043	0.56	1.4	0.94	−0.00003	0.89
Maximum of the ICA IMT >1.5 mm	1.14	0.77	1.68	0.51	0.0013	−0.0016	0.0042	0.89	1.5	0.32	−0.00009	0.68
Mean of the maximum ICA IMT, mm	1.14	0.89	1.47	0.28	0.0002	−0.0036	0.0040	0.10	2.4	0.14	0.00058	0.17

HR indicates hazard ratio; NRI, net reclassification improvement; IDI, integrated discrimination improvement; ICA, internal carotid artery; IMT, intima‐media thickness.

Mean of the maximum CCA IMT was not associated with either CVD or stroke but was associated with CHD with a hazard ratio of 2.10 (95% CI 1.30–3.40) and an increase in C‐statistic of 0.0055 (95% CI 0.001–0.0100). It remained significant for only 2 of the plaque metrics when these were in the model (presence of any plaque and presence of plaque causing >25% diameter narrowing at the bulb). For these 2 plaque definitions, the NRIs were 5.7% (*P*=0.010) and 4.7% (*P*=0.014) respectively and similar to the NRIs without common carotid IMT in Table 5.

## Discussion

In our multiethnic cohort, carotid artery plaque metrics added incremental value for the prediction of coronary artery disease events. Carotid plaque metrics were independently associated with first‐time CHD events after adjusting for Framingham Heart cardiovascular risk factors, increased the C‐statistics of the Cox proportional hazards prediction models, and significantly increased NRIs. These metrics include the purely subjective evaluation of the degree of carotid artery narrowing,^10^ the maximum IMT measured as the largest value obtained on any of the ICA images^8^ or averaged over all possible measurements,^15^ and a combination of presence of plaque >0% and a far wall ICA IMT >1.5 mm.^11^ With regard to the broader endpoint of incident CVD, only 1 metric, mean of the maximum ICA IMT, significantly improved risk prediction over what was achieved by a model with Framingham risk factors alone. None of the plaque metrics added significantly to the prediction of incident stroke.

Given the multiethnic nature of our cohort, we adjusted for race/ethnicity in our models. Although this approach can adjust for the effects of race/ethnicity, we could not study separate models for each ethnicity due to the smaller numbers of participants in each stratum.

We were not able to include other plaque metrics due to their large number and focused on ones that (1) had shown associations with events in other cohorts, (2) were familiar to us, and (3) were easily extracted from our dataset. This is a limitation. We were not able, at this time, to include measurements of plaque area.^25–26^ It is possible that these measures might have greater predictive power for cardiovascular events or stroke than those we analyzed.

The presence of any perceivable lesion in the ICA has shown some association with prevalent cardiovascular events when stratified as 1% to 24%, 25% to 49%, and >50% diameter narrowing in the Cardiovascular Health Study (CHS).^10^ There are also reports that subjectively scored presence of plaque, and often the number of plaques, are associated with cardiovascular events.^9,27^ We did not count the number of lesions as part of our analysis. This is a limitation of the use of a dichotomous plaque variable that focuses on the largest plaque seen during a carotid artery examination. As to generalizability of our results, the approach used to grade presence and severity of plaque in MESA is the same as in the CHS protocol.^10,28^

The plaque metric derived from the Atherosclerosis Risk in Communities study (ARIC)^7,11^ is a combination of a subjective judgment on the presence of a protrusion into the artery lumen or a far wall maximum IMT >1.5 mm. The derivation of this metric in our dataset might be limited by differences in the ARIC and MESA protocols. ARIC specifically looked at only the far wall of the ICA and CCA, on one view of the ICA and 3 views of the CCA for each side.^29^ We only had one view of the CCA to measure common carotid maximum IMT compared with 3 in ARIC, whereas we had 3 views to measure the internal IMT versus the 1 view for ARIC. In addition, ARIC reported a low completeness rate for IMT values taken in the ICAs.^30^ We used the definition of plaque present/absent as presented in the ARIC manual^29^ to be similar to our own, that is, any protrusion. It is possible that the ARIC readers used a higher threshold for defining a protrusion. It is also possible that we visualized more lesions since we included the near wall of the ICA, whereas ARIC did not. This should be given consideration given the large number of missing ICA IMT values in ARIC, up to 57%,^31^ whereas in MESA only 185 measurements were missing of the 6814 cohort members.

The mean of the maximum IMT as measured in the CHS is associated with cardiovascular outcomes defined as a combination of MI and stroke.^15^ The MESA protocol is essentially identical to the CHS protocol, and as such we believe that we replicated the measurement process accurately.

At first glance, the process used to determine the maximum ICA IMT in MESA^14^ appears slightly different than the one used in the Framingham study.^13^ However, in both cases, the sonographers were trained to acquire at least 1 image that best showed the thickest IMT. In MESA, the sonographer used the view closest to 1 of the 3 required images taken in the anterior, lateral, or posterior projections. In Framingham, the sonographer picked a view in either the anterior or posterior projection after taking a standard lateral image. The derived plaque variables, maximum ICA IMT and maximum ICA IMT >1.5 mm, should therefore be very similar.

A major strength of our study is the absence of prevalent CVD at baseline. As such, our results might, on the long term, be applicable to the risk stratification and primary prevention of CHD. Three of our metrics depend on the ability of a trained reader for detecting and qualitatively grading early carotid lesions. This, we believe, is achievable by most trained sonographers who have experience with clinical carotid artery examinations. The presence of lesions causing <50% diameter narrowing is already considered as an element of the clinical ultrasound examination.^32^ Discerning small from moderate carotid lesions was motivated by potential applicability to the clinical carotid ultrasound examination and achieved by reviewing static carotid images as well as a video recording made during a transverse sweep of the neck.

Limitations to the general applicability of our findings might be our 98.4% data completeness rate for ICA IMT measurements (6629/6739) and a 98.6% success rate for estimating plaque severity (6716/6739) in the 6739 carotid ultrasound examinations performed (of 6814 MESA participants). While the rate of IMT measurements is significantly higher than for ARIC^30^ and the Rotterdam studies,^33^ they are similar to those reported in the Framingham Heart Study^13^ and the CARDIA study.^34^

The emphasis of our investigation was to compare the applicability of different plaque metrics estimated from carotid ultrasound images as a means of predicting future cardiovascular events. As such, we did not focus on the role of any particular risk factor but rather on the incremental increase in C‐statistic when a plaque metric was added to a baseline model that included only Framingham risk factors. While all of the selected plaque metrics were associated with incident CVD, all but 1 of these metrics increased the C‐statistic of the models over and above the Framingham risk factors and only 1, the mean of the maximum IMT, increased the NRI. We further broke down our composite outcome of CVD into its constituents: first‐time events due to coronary artery disease and stroke. The associations with stroke were weak, possibly due to the low incidence of events or simply to the fact that these metrics do not predict incident stroke as well as they predict incident CHD. The associations of all plaque metrics were consistently strong for CHD events, since each was an independent predictor of events, was associated with an increase in the C‐statistic when added to a model with risk factors and, with the exception of 1 metric, incremented the NRI between 4.2% and 7.0%. The latter finding is consistent with the reported by Polak et al^8^ although the NRIs are lower. We attempted to replicate the recently findings from ARIC that showed that plaque measurements and common carotid IMT in combination add to risk prediction of coronary artery disease events. While we have found that the CCA IMT was a predictor of coronary artery disease events, it only seemed to contribute to events when paired with a subjective measurement of plaque and not with a quantitative measurement of ICA IMT. This is consistent with recent ARIC observations showing that subjective plaque observations and CCA IMT contribute to the prediction of CHD events.^31^

Adding clinic site and reader to the prediction models to account for local differences during image acquisition at the 6 MESA clinic sites and for interreader variability when estimating the degree of carotid artery stenosis and measuring IMT did not alter our results.

In conclusion, we compared the predictive value of 6 different metrics of carotid artery plaque and found that all were independent predictors of CHD events, increased the C‐statistics, and all but 1 increased the NRI. In addition, 1 plaque metric, the mean of the maximum IMT, incrementally added to the prediction of incident CVD beyond what can be achieved with traditional Framingham risk factors.
